# Know How to Regrow—Axon Regeneration in the Zebrafish Spinal Cord

**DOI:** 10.3390/cells10061404

**Published:** 2021-06-06

**Authors:** Vasiliki Tsata, Daniel Wehner

**Affiliations:** 1 Experimental Surgery, Clinical and Translational Research Center, Biomedical Research Foundation Academy of Athens, 11527 Athens, Greece; 2 Max Planck Institute for the Science of Light, 91058 Erlangen, Germany; 3 Max-Planck-Zentrum für Physik und Medizin, 91058 Erlangen, Germany

**Keywords:** zebrafish, spinal cord injury, axon regeneration, functional recovery

## Abstract

The capacity for long-distance axon regeneration and functional recovery after spinal cord injury is poor in mammals but remarkable in some vertebrates, including fish and salamanders. The cellular and molecular basis of this interspecies difference is beginning to emerge. This includes the identification of target cells that react to the injury and the cues directing their pro-regenerative responses. Among existing models of successful spinal cord regeneration, the zebrafish is arguably the most understood at a mechanistic level to date. Here, we review the spinal cord injury paradigms used in zebrafish, and summarize the breadth of neuron-intrinsic and -extrinsic factors that have been identified to play pivotal roles in the ability of zebrafish to regenerate central nervous system axons and recover function.

## 1. Introduction

The inability of severed axonal fibres to regrow across the lesion site after spinal cord injury (SCI) prevents recovery of locomotor function and results in permanent functional deficits in humans. The severity and level of the injury define a broad range of symptoms. These include partial to complete loss of sensory and locomotor function, sexual, bowel and bladder function impairments, heart, breathing and blood pressure dysregulation and chronic pain. To date, no effective treatment exists that can reverse the pathology, which has debilitating consequences for affected individuals [1]. Hence, enabling long-distance growth of spinal cord connections after injury to achieve substantial functional recovery remains one of the greatest challenges in regenerative medicine. During embryonic and neonatal stages some mammals such as mice and opossum [2,3,4], birds [5] and anuran amphibians [6] can regrow central nervous system (CNS) axons. However, with the possible exception of the African spiny mouse [7], in all of these cases developmental age alters the regenerative capacity of the CNS, which sharply declines after birth and is eventually lost. Contrary to mammals, many aquatic species including urodele amphibians (axolotl, newt) [8,9] and fish such as carp [10], eel [11], lamprey [12,13], goldfish [14] and zebrafish [15] exhibit a remarkable regenerative capacity that persists to adulthood and throughout the CNS, including the brain [16], spinal cord [15] and the visual system [17,18,19]. Although a holistic understanding of the mechanisms underlying successful CNS regeneration in these species is still elusive, important cellular and molecular players have been identified. Deepening insights on neuron-intrinsic and -extrinsic factors facilitating functional regeneration in regeneration-competent species could be potentially leveraged for the development of novel therapeutic approaches in humans. In this review, we focus on zebrafish, which due to their genetic amenability and excellent optical properties at larval stages, have provided a mechanistic insight on how the spinal cord can be successfully repaired after injury. In particular, we will focus on the events identified to facilitate axonal regrowth of axotomized neurons—the pre-requisite for functional restoration after SCI. It is important to note that besides axon regeneration, zebrafish respond to SCI with robust regenerative neurogenesis, which was expertly reviewed elsewhere [20,21].

## 2. Axon Regeneration and Functional Recovery after SCI in Zebrafish

Similar to mammals, following an injury to the spinal cord, zebrafish are initially paralyzed caudal to the lesion site. However, they successfully recover most of their swimming activity within a few days (larva) or weeks (adult) [22,23,24,25]. It is well documented that restoration of swimming function critically depends on the regrowth of axonal projections across the lesion site. When axonal regrowth was experimentally hindered through creation of a physical barrier at the lesion site, functional recovery was absent [22]. Similarly, zebrafish larvae that failed to form axonal connections (bridge) between the severed spinal cord ends exhibited markedly worse recovery of swimming distance [26]. Re-transection of regenerated spinal cords abolished the restored swimming activity in both systems, underlining the necessity of axon regeneration for functional recovery [24,27].

### Differential Regenerative Capacity of Zebrafish Axons

Although much less is known about the composition of regrowing axons in larval as compared to adult zebrafish, research over the last few decades has revealed a differential regenerative capacity among different neuronal subtypes. This depends on the level of axotomy (distance from the soma), the size/length of the neuronal projection, as well as the direction of the axonal tract (descending, ascending). Early experiments using the neural tract-tracing technique (anterograde and retrograde axonal tracing) showed that spinal cord transection severs descending axons from 20 distinct brain nuclei, ascending axons to the brainstem as well as intraspinal descending and ascending axons [15]. The majority of brain nuclei regrow axons beyond the transection site into the distal spinal cord [15,22,28,29]. However, brain nuclei are distinct in their regenerative capacity such as the nucleus of the medial longitudinal fascicle (nMLF) or the nucleus of the lateral lemniscus, which can be viewed as good and poor regenerators, respectively [30]. Although greatly altered as compared to pre-injury levels, dopaminergic (TH1^+^) and serotonergic (5-HT^+^) terminals are also re-established [27]. Finally, intraspinal neurons have been reported to extend axons across the lesion site [31], which includes a very low number of ascending tracts [30]. In larval zebrafish, a systematic analysis on the regenerating axonal subtypes after SCI is currently missing as studies have mainly used pan-neuronal markers to label neurites. However, both long descending projections from the brainstem such as the nMLF axons, which control swimming function [32], as well as axons from intraspinal neurons (e.g., *mnx1*^+^, *vsx2*^+^), appear to robustly contribute to the axonal bridge (D. Wehner, T. Becker, C. G. Becker; unpublished observations; [33]). Interestingly, while reticulospinal Mauthner axons seem to fail to regenerate in the adult zebrafish [15], their robust regeneration was reported by some studies in larval animals to depend on the level of axotomy along the rostro-caudal axis [34,35,36]. Of note, with the exception of the Mauthner axons [34], which have been directly linked to a short latency escape response in larval animals [37], the contribution of individual axons to functional recovery after SCI remains to be investigated. Collectively, a great body of research has demonstrated that larval and adult zebrafish axons successfully regenerate after SCI. Although anatomical regeneration of the spinal cord is imperfect [31,38,39,40], regrown axons innervate appropriate targets, which suffices for functional restoration [22,24].

## 3. A Breadth of Zebrafish SCI Paradigms

SCI models are mainly categorized according to: (i) the injury mechanism, (ii) the site of the injury and, (iii) the model organism used. In mammals, SCI paradigms usually involve an acute injury such as contusion, compression, dislocation and transection or a graded damage, such as distraction (stretching) and chemical injury, while injury levels vary from lumbar, to cervical and thoracic level. Several injury models have been also developed in zebrafish. Yet, the difference to mammals is robust axonal regrowth and functional recovery.

### 3.1. Mechanical Lesion

Among the injury paradigms frequently used is a mechanical lesion. In larval zebrafish the spinal cord is completely transected at the level of the urogenital pore through an incision using either sharp glass [36,41] or an injection needle [24,42]. Alternatively, a stab lesion has been employed [26,43]. Lesioning abolishes swimming function, which is restored within a few days after the injury [24,25].

In adult zebrafish, a mechanical lesion to the spinal cord is inflicted by a complete transection, approximately 5 mm caudal to the brainstem-spinal cord junction, which corresponds to a midthoracic level injury [15]. As in the larval model, this injury paradigm leaves no axons spared at the lesion site and provides an optimal platform to study axon regeneration. Alternatively, a crush injury in which forceps compress the spinal cord at the level of the 15–16th vertebrae, causes axonal degeneration without resulting in complete transection and assimilates an injury mode occurring in humans [44]. In both cases, fish are paralyzed caudal to the injury site, yet recovery of swimming activity typically occurs within 6–8 weeks post-lesion [22,23].

### 3.2. Alternative Lesion Paradigms

In addition to mechanical lesions, a variety of cell ablation paradigms have been used to investigate axon regeneration in zebrafish. The Nitroreductase (NTR)/Metronidazole (MTZ) system [45] provides a pharmacogenetic platform to selectively ablate spinal neurons within hours of treatment and to monitor their fast regeneration within 2 days [24]. To overcome the dependence of such an approach on the availability of tissue-specific promoters, an optogenetic system was developed that utilizes a photoactivatable GAVPO transactivator element to light-inducible drive expression of the cytotoxic ion channel variant M2^H37A^ [46]. Furthermore, electroablation has been used to induce localized spinal damage [47]. The latter relies on the application of an electrical pulse and allows the investigation of axon regeneration after neurectomy. Finally, spinal cord lesions and selected axotomy of trigeminal sensory axons have been reported utilizing a 2-photon microscope with a focused high-power laser [48,49]. Overall, a plethora of different injury paradigms have confirmed the high regenerative capacity of the zebrafish spinal cord and provide a variety of systems to dissect the mechanistic underpinnings of successful axonal regrowth. Due to the rapid regeneration time and optical accessibility for live imaging, the larval model offers an ideal platform to study the dynamics of cellular interactions in the injured spinal cord. Adult fish are better suited when analyzing processes that are not fully developed in larvae, such as the adaptive immune response, or when more complex behavioral readouts are needed.

## 4. Neuron-Extrinsic and -Intrinsic Factors Define Successful Axon Regeneration in Zebrafish

**Figure 1:**
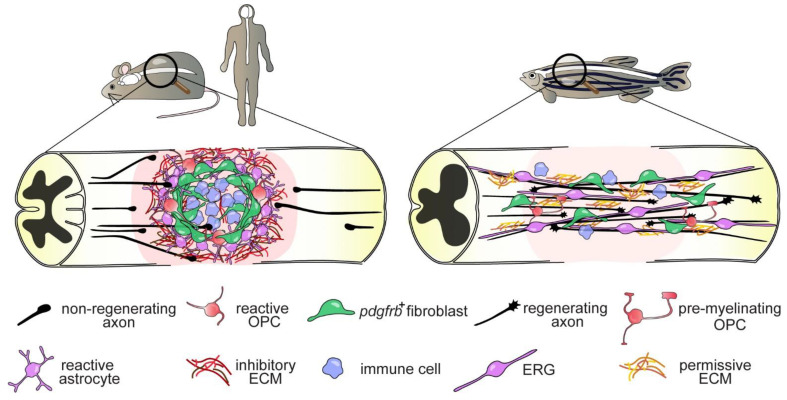
Differential regenerative capacities of the mammalian and zebrafish spinal cord. In mammals, a complex injury scar is formed by invading immune cells and *pdgfrb*^+^ fibroblasts, as well as reactive OPCs and astrocytes, which constitutes a hostile environment to axon growth. Although these cell types respond in a similar manner to SCI in zebrafish, they do not contribute to the formation of an injury scar. Instead, reactive cells promote axonal regrowth (bridging) across the lesion site. Shown are major target cells underlying the different fate in either species. Abbreviations: OPC, oligodendrocyte progenitor cell; *pdgfrb*^+^, platelet-derived growth factor receptor β-positive; ECM, extracellular matrix; ERG, ependymo-radial glia.

SCI in mammals results in: (i) neuronal and glial cell loss, (ii) activation of the immune system and, (iii) rapid recruitment of non-neuronal cells, such as fibroblasts, to the lesion site. The cellular events that follow the initial damage caused by the primary impact create a regeneration-limiting zone [50,51] that further develops and expands over time, inhibiting axonal regrowth and functional recovery (Figure 1). To date, several neuron-intrinsic (e.g., cytoskeleton disorganization, impaired transport of retrograde signals) and -extrinsic (“environmental”) factors (e.g., inflammation, scarring) have been identified to contribute to the failure of axonal regrowth after SCI in mammals. In sharp contrast, zebrafish CNS axons possess a high intrinsic capacity for regenerative growth, which in combination with a permissive environment, promotes axon regeneration beyond the lesion site and enables functional recovery [52] (Figure 1).

### 4.1. Immune System

**Figure 2:**
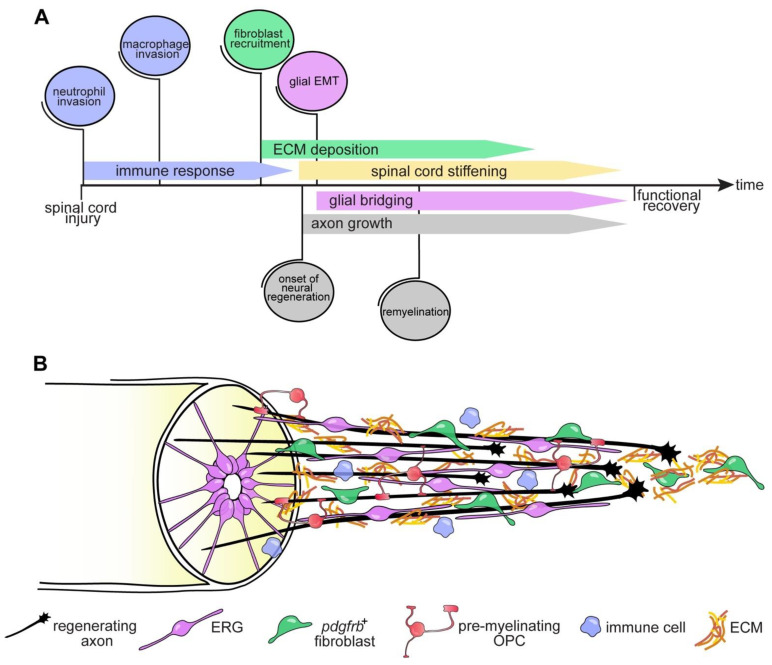
Cellular responses and events facilitating axon regeneration after SCI in zebrafish. An injury to the zebrafish spinal cord induces an immediate yet transient immune response mediated by activation of both resident microglia as well as invasion of peripheral neutrophils and macrophages. This is followed by the recruitment of *pdgfrb*^+^ fibroblasts that deposit a growth-permissive ECM. Axons navigate the ECM-rich lesion environment. ERGs undergo an epithelial-to-mesenchymal transition (EMT) and participate in the formation of a neural tissue bridge that reconnects the severed spinal cord. Reactive OPCs differentiate into pre-myelinating OPCs that will remyelinate regrowing axonal fibres. The roles of individual reactive cells are described in detail in the main text. The sequence of events and the spatial relationship of major cellular components involved in axonal regrowth are summarized in (**A**) and (**B**), respectively. Note that no specific timepoints are given as the schematic view summarizes recent findings obtained from studies in larval and adult zebrafish. Abbreviations: EMT, epithelial-to-mesenchymal transition; ERG, ependymo-radial glia; *pdgfrb*^+^, platelet-derived growth factor receptor β-positive; OPC, oligodendrocyte progenitor cell; ECM, extracellular matrix.

**Figure 3:**
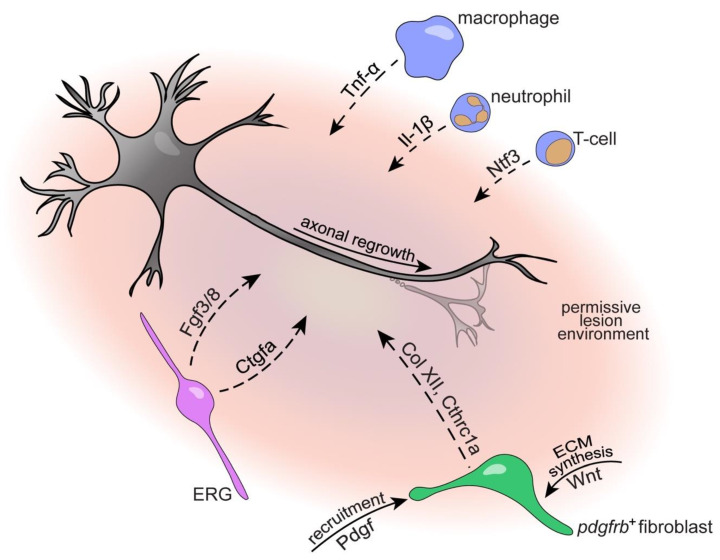
Key molecular and cellular players contributing to the axon growth-permissive lesion environment after SCI in zebrafish. ERGs secrete growth factors (Fgf, Ctgf), *pdgfrb*^+^ fibroblasts deposit growth-conducive ECM (Col XII, Cthrc1a) molecules and immune cells dynamically control inflammation promoting axon regeneration. The roles of individual cells and contributing factors are described in detail in the main text. Arrows indicate direct interaction of secreted factors with target cells, dashed arrows indicate indirect interaction or not shown to be direct. Abbreviations: Tnf-a, tumor necrosis factor α; Il-1β, interleukin-1β; Ntf3, neurotrophin 3; ECM, extracellular matrix; *pdgfrb*^+^, platelet-derived growth factor receptor β-positive; Col XII, collagen type XII; Cthrc1a, collagen triple helix repeat containing 1a; Pdgf, platelet-derived growth factor; ERG, ependymo-radial glia; Ctgfa, connective tissue growth factor; Fgf3/8, fibroblast growth factor 3/8.

Activation of resident microglia and the release of pro-inflammatory cytokines such as interleukins (IL) IL-1b, IL-6 and tumor necrosis factor α (TNF-α) are among the early events occurring within the first minutes to hours after SCI in mammals [53,54]. Together with the invasion of peripheral neutrophils, macrophages and T-lymphocytes that infiltrate the injury site in a time-dependent response [55], the persistent activation of the immune system leads to a prolonged inflammatory phase that participates in lesion propagation and contributes to a hostile environment that inhibits axonal regrowth [56]. Substantial evidence indicates that in the zebrafish spinal cord, acute inflammation is necessary and sufficient for axonal regrowth [24,57,58] (Figure 1). After injury, a timed response of consecutively appearing neutrophils and macrophage/microglia occurs. During this phase, immune cells are found to be closely associated with and/or engulf cellular and myelin debris, indicating clearance of the latter [29,57]. Interestingly, increasing cellular debris levels in the lesion site through interference with phagocytosis did not impair axon regeneration, similar to what has been suggested for the regeneration of the optic nerve in mice [59]. This suggests that phagocytosis and removal of cellular debris are of rather minor importance to axon regeneration [57]. Suppression of microglia, macrophage and neutrophil responses by dexamethasone treatment impaired regeneration, indicating a promoting role of innate immune cells in axonal regrowth and functional recovery after SCI [57,58]. Analyses of interferon regulatory factor 8 (*irf8*)-deficient mutants that lack macrophages and microglia but not neutrophils, revealed a biphasic role of inflammation on axon regeneration. A transient upregulation of proinflammatory cytokines Il-1β and Tnf-α is beneficial rather than detrimental to the regenerative capacity of axons [57]. This proinflammatory phase needs to be tightly controlled though, as its prolongation by genetic depletion of macrophages or loss-of-function of the anti-inflammatory cytokine transforming growth factor beta-1 (*tgfb1a*), inhibits regeneration [57,60]. Of note, in larval zebrafish, microglia appear to play a rather negligible role in promoting axon regeneration as compared to peripheral macrophages. In addition to the innate immune response, the adaptive immunity is crucial for axon regeneration in adult zebrafish. T regulatory cells infiltrate the injured spinal cord and secrete regeneration-promoting factors, such as neurotrophins (neurotrophin-3; Ntf3), and their targeted ablation impairs axonal growth and recovery of swimming activity [61]. Together, the precise timing and regulation of the immune response after SCI critically contributes to the creation of a regeneration-permissive environment in zebrafish and highlights potential components of the injury-induced cellular reactions that may be targeted in non-regenerating mammals (Figure 2 and Figure 3). Notably, the immune system-derived factor TNF-α, which was identified as critical for regeneration in zebrafish [57], has also been highlighted as target for therapeutic intervention after SCI in humans [62]. This supports the relevance of the zebrafish model for translational spinal cord regeneration research.

### 4.2. Oligodendrocytes and Oligodendrocyte Progenitor Cells

In addition to their function as insulators and their role in signal transduction through myelin wrapping, oligodendrocytes support axonal survival through structural, metabolic and trophic support [63,64,65,66]. Furthermore, oligodendrocyte progenitor cells (OPCs) communicate and interact with axons bidirectionally and dynamically throughout life via synaptic connections [67], allowing the modulation of OPC biology and myelination by neuronal activity [68,69]. Although OPCs are capable of myelination after SCI [70] and alleviation of locomotor deficits can occur in the absence of complete re-myelination [71], the insufficient replenishment of lost oligodendrocytes is considered a contributing impediment to axon regeneration after SCI. Although a significant loss of mature, myelinating oligodendrocytes is also observed after SCI in adult zebrafish, OPCs apparently largely survive the primary and secondary damage [38,40]. Importantly, OPCs differentiate to re-establish the oligodendrocyte population within two weeks post-injury and contribute to re-myelination of regenerated axonal fibres [38,40]. Negligible death of OPCs has also been reported after SCI in larval zebrafish [24], supporting the notion that cellular responses to SCI are conserved between larval and adult stages.

The formation of synaptic-like structures between presumptive OPCs (NG2^+^ cells) and axonal terminals that entrap neurites and thereby halt their growth, represents an additional obstacle to mammalian CNS axon regeneration [72,73]. In addition, a variety of oligodendrocyte lineage-derived molecules, specifically myelin membrane bound proteins, including oligodendrocyte-myelin glycoprotein (OMgp), myelin-associated glycoprotein (MAG), neurite outgrowth inhibitor (Nogo) and chondroitin sulfate proteoglycans (CSPGs) such as NG2 (CSPG4), inhibit axonal regrowth by destabilizing growth cones that become dystrophic and eventually degenerate [74]. Interestingly, zebrafish Reticulon (Rtn)/Nogo family proteins appear to be less growth inhibitory, as axons of retinal ganglion cells regenerated successfully in the presence of the zebrafish ortholog Rtn4b in vitro and in vivo [75]. Furthermore, spinal cord transection leads to increased expression of the immunoglobulin superfamily molecule protein zero (P0), an important component of myelin formation in white matter axonal tracts, suggesting a prominent role for P0 in myelination and promotion of axon regeneration in zebrafish [76]. Concomitantly, transcriptional profiling of zebrafish OPCs revealed upregulation of genes coding for extracellular matrix (ECM) and ECM-remodeling enzymes after SCI, including connective tissue growth factor (*ctgfa*), semaphorin 3c (*sema3c*) and tenascin-c (*tnc*) but not inhibitory *cspg4*. This suggests a role for zebrafish OPCs in supporting axon regeneration by secreting growth-permissive ECM [40]. Together, these data point to a beneficial role of oligodendroglia lineage cells to axon regeneration after SCI in zebrafish (Figure 1 and Figure 2). Future studies need to investigate how the cellular response of oligodendroglia lineage cells precisely affect axonal regrowth and signal propagation, key events for the functional restoration and fine coordination of locomotor activity after injury.

### 4.3. The Glial Cell and Fibroblast Response to SCI

The formation of an injury scar, often exhibiting fluid-filled cystic cavities [77,78], is a major barrier to axon regeneration across the lesion site in the mammalian CNS. Consisting of different regions with distinct cellular and molecular signatures [79,80], the scar is complex and involves a fibrous core in which invading fibroblasts deposit a dense meshwork of ECM that is inhibitory to axonal growth [81]. Surrounding the fibrous core, the glial component of the scar consist of reactive astrocytes, NG2^+^ presumptive OPCs and cells of the immune system that migrate to the lesion site upon activation [82]. Although scar formation is crucial in stabilizing the expansion and spread of uncontrolled tissue damage and inflammation [78] to ensure overall survival [83], and the extent to which astrocytes inhibit axon regeneration is currently debated [84], local tissue re-modelling creates a barrier to axonal regrowth [79] (Figure 1).

#### 4.3.1. Ependymo-Radial Glia

The glial component of the scar in mammals is composed of cells of the ependymal niche, which are activated after injury, proliferate and generate scar-contributing astrocytes [85,86]. Although limiting secondary damage, glial cells are thought to produce inhibitory ECM molecules, including CSPGs [87]. In zebrafish, Glial fibrillary acidic protein (GFAP)^+^ astrocyte-like glia, which correspond to ependymo-radial glia (ERGs) that have their soma located at the central canal and extend radial processes that span the parenchyma, do not separate the injury site from the spared tissue (Figure 1 and Figure 2). Instead, ERGs undergo a Hippo signaling-controlled and Twist1-mediated epithelial-to-mesenchymal transition (EMT), elongate their processes and, together with regrowing axons, participate in the formation of the neural tissue bridge that reconnects the severed spinal cord [26,31,88]. As ERGs do not contribute to the formation of an injury scar, a body of research has focused on the identification of factors promoting glial bridging during regeneration of the zebrafish spinal cord. Yet, their specific role in axon regeneration has been subject to controversy. Glial bridges have been proposed to provide a substrate for regenerating axons to cross the lesion site [31]. Although a systematic ultrastructural analysis is lacking in zebrafish, live observations of glial and neuronal processes in larval animals [26], histological analysis in the eel [89] and ultrastructural analysis of the adult goldfish [90] provided evidence that axonal fascicles precede glial cells when navigating the lesion site. Importantly, axonal bridging was also established when GFAP^+^ glia were selectively ablated using the NTR/MTZ system, indicating that glial bridging is dispensable for axonal regrowth across the spinal lesion site, at least in larval zebrafish [26]. Although, it remains to be demonstrated whether adult fish fully recapitulate the observations made in larvae, these data argue for a different function of glial cells in supporting axon regeneration in zebrafish. During development and homeostasis, glial cells are a major source of trophic factors that promote neuronal cell migration, growth and survival [91]. Indeed, after SCI in zebrafish a heterogeneous cell population, that includes ERGs, secretes Ctgfa and its loss in *ctgfa* mutants impaired axon regeneration and functional recovery; an effect that can be rescued by systemic over-expression of *ctgfa* or application of recombinant human CTGF [23]. However, as the cells that are competent to respond to Ctgfa by means of a ligand-receptor interaction are unknown, it remains to be investigated whether Ctgfa acts on neurons to promote axonal regrowth directly or indirectly through control of ECM deposition in the lesion site—a well-established function of CTGF in the context of injury [92,93] (Figure 3). Similarly, ERGs and neurons secrete fibroblast growth factors (FGF) 3 and 8a [31] (Figure 3). FGF ligands presumably activate the FGF pathway in an autocrine manner as target genes (*spry*, *pea3* and *erm)* are expressed in ERGs and neurons [31]. Systemic genetic or pharmacological interference with FGF signaling inhibited both glial and axonal bridging [31,94]. Thus, formation of a tissue bridge after SCI requires FGF pathway activity in neural cells. As cell type-specific manipulations can identify surprising complexity during tissue regeneration [95], future work using ERG-specific manipulations [26,96] could provide novel insight on a potential direct link between glial cell-derived molecular cues and axon regeneration.

#### 4.3.2. Fibroblasts

The deposition of ECM by invading non-neural cells is considered a major contributor to the growth non-permissive injury scar that axons encounter upon CNS pathologies in mammals [97] (Figure 1). Perivascular cells have been identified as a primary source of scar-forming fibroblasts after SCI [98,99]. Attenuating fibrosis by interference with fibroblast generation or migration [100,101,102], or depriving the lesion site of ECM components by inhibition of collagen biosynthesis and deposition [103,104] or enzymatic inactivation [105], enhances permissiveness of the scar. This underscores the fibrous scar as a target for therapeutic interventions after SCI.

Similar to mammals, fibroblasts infiltration and prominent ECM deposition have been described after SCI in zebrafish [26,106], goldfish [107] and other regenerating vertebrates [8]. In zebrafish, a fibroblast population of perivascular and myoseptal origin that enters the lesion site can be identified by the expression of the platelet-derived growth factor receptor (Pdgfr) β, similar to what has been described in the mouse spinal cord [98,99]. Thus, the initial cellular response to SCI appears to be largely conserved between zebrafish and non-regenerating vertebrates, although additional fibroblast subtypes likely exist in both species that contribute to the lesion site population [108]. Different to mammals however, in zebrafish, *pdgfrb*^+^ fibroblasts are required for axon regeneration and functional recovery while cell autonomous signaling through the Pdgf receptor (Pdgfr) has been identified as a molecular cue controlling their recruitment [106] (Figure 2 and Figure 3). Interestingly, Pdgf signaling also appears to be a conserved chemoattractant for fibroblasts contributing to organ regeneration in other species [109]. Yet it is unknown whether signaling through the Pdgfr may identify a target for alleviating perivascular cell-derived scarring in mammals. The regeneration-promoting effect of zebrafish *pdgfrb*^+^ fibroblasts after SCI has been attributed to the secretion of a regeneration-permissive ECM that is enriched in growth-promoting (e.g., collagen type XII (Col XII), collagen triple-helix repeat containing 1a (Cthrc1a), Tenascin-C (TnC)) and deprived of growth-limiting matrix molecules (e.g., Lumican (Lum), Microfibril Associated Protein 2 (Mfap2), Periostin (Postn), collagen type IV (Col IV)) [106]. Thus, the specific composition of the fibroblast-derived ECM appears to define a key difference between zebrafish and mammalian fibroblast response and regenerative outcome upon SCI. Future studies will need to establish the detailed function of specific matrix components in promoting axonal regrowth in zebrafish as well as to systemically map the differences in ECM composition between regenerating and non-regenerating species. Although the cues controlling the composition of the fibroblast-derived ECM remain to be identified, Wnt/ß-catenin (Wnt) signaling has already been shown to play a decisive role in zebrafish. Wnt signaling promotes regeneration in larval and adult zebrafish [110,111] by acting on *pdgfrb*^+^ fibroblasts to control expression of pro-regenerative type XII collagen, which is necessary and sufficient for axon regeneration [26] (Figure 3). Interestingly, Wnt pathway activity in fibroblasts is required for both glial and axonal bridging, suggesting that glial cells and regrowing neurites utilize ECM as growth substrates to cross the lesion site [26].

Increasing evidence indicates that the injury scar exerts its inhibitory function on axonal regrowth not only through its biochemical composition but also acts as a mechanical barrier due to the altered elastic stiffness of the local microenvironment [112]. Indeed, axons interact with their environment not only chemically but also mechanically [113]. However, very little is known about the role of mechanical signals involved in axonal regrowth. Furthermore, the contribution of ECM to the mechanical properties of the lesion environment as well as that of local tissue mechanics to regenerative success remains poorly investigated. Recently, atomic force microscopy-enabled nanoindentation revealed a stiffening of the zebrafish spinal cord during regeneration, which contrasts the compliant signature of the growth-inhibitory scar tissue found in mammalian CNS lesions [114]. Future studies investigating the differences in mechanical properties of the lesion environment between regenerating and non-regenerating species as well as the identification of their determinants will reveal previously unappreciated neuron-extrinsic factors contributing to regeneration success versus inhibitory scarring. Such information will aid the development of biomaterial-scaffold strategies used to stimulate axon regeneration after SCI in mammals.

## 5. Intrinsic Regenerative Capacity

In addition to the regeneration barrier created by the hostile lesion environment, a battery of neuron-intrinsic molecular pathways, that are active during development yet fail to reactivate and initiate a successful growth response after injury, determine the limited capacity of mammalian axons to regrow [115].

SCI disrupts axonal homeostasis due to physical and metabolic perturbations. These include rupture of the axon plasma membrane, rapid elevation of intra-axonal Ca^2+^ concentration, destabilization of actin and microtubule cytoskeletal network and disorganization of intra-axonal organelles. In mammals, these events do not result in the activation of gene-expression programs necessary for growth cone formation and neurite extension [116,117]. In contrast, in the zebrafish CNS, axotomy-induced signals are successfully translated into a regeneration response. The upregulation of the major vault protein (*mvp*), constituent of the ribonucleoprotein complex, during the acute phase after SCI has been proposed as a potential regulator of cell survival under conditions of cellular stress, acting as an anti-apoptotic factor [118]. In *mvp* null mutant zebrafish cell death was enhanced after SCI, although little effect on axon regeneration could be observed. Surprisingly, *mvp* knockdown by use of morpholino oligonucleotides was reported to perturb axonal regrowth and functional recovery, suggesting a direct role of Mvp protein for successful axon regeneration [119]. Future studies will need to clarify the precise function of Mvp in spinal cord regeneration. The levels of activating transcription factor 6 (*atf6*), a type-II transmembrane protein in the endoplasmic reticulum and a major stress sensor, as well as its target genes binding immunoglobulin protein (*bip*) and C/EBP homologous transcription factor protein (*chop*), were also tightly regulated after SCI in adult zebrafish. *atf6* knockdown inhibited axon regeneration and delayed recovery of swimming activity, suggesting a role for endoplasmatic reticulum homeostasis to axonal protection and regeneration [120]. Furthermore, brainstem and intraspinal neurons upregulate cell recognition and guidance molecules such as Growth Associated Protein 43 (GAP-43), L1.1, L1.2 and Semaphorin 4D, all of which support axon regeneration and functional recovery [22,28,121]. Intriguingly, the upregulation of growth-promoting molecules depended also on the distance of the lesion to the neuronal soma, highlighting an intrinsic spatially-controlled regulation of axon regeneration [28]. Similarly, the proximity of the injury site to the soma determines the regeneration of the Mauthner axon. Regrowth occurs following injuries between 10% and 50% of the total axon length but is very poor when axotomy is located both close as well as distant to the soma [34]. Regeneration capacity of the Mauthner axon could be enhanced by increasing cyclic adenosine monophosphate (cAMP) levels and mitochondrial transport [36,122], highlighting cellular energetics as a strategy to promote axonal regrowth and functional recovery after SCI [123,124]. The initiation of sprouting, as well as the selection of the optimal trajectory to establish functional synapses, is of crucial importance for successful pathfinding and functional axon regeneration. Indeed, in the larval and adult zebrafish, regenerating axons grow through novel pathways, including the grey matter [26,29,47]. Interestingly, experiments in axotomized Mauthner axons revealed that axons cross the lesion site by first initiating multidirectional sprouting and then correcting their route in a ubiquitin ligase Pam/Highwire/Rpm-1 (PHR)-dependent manner. This process was largely dependent on the tight regulation of actin polymerization by cytoplasmic FMR1 interacting protein 2 (Cyfip2) and c-Jun N-terminal kinase (JNK) signaling pathways [125], implicating growth cone extension for axon regeneration. While knowledge on intrinsic pro-regenerative factors in spinal-projecting neurons is still limited, previous studies on the optic nerve, which likewise robustly regenerates in zebrafish, have resulted in additional insights and are reviewed in detail elsewhere [52].

## 6. Conclusions and Outlook

Zebrafish have greatly advanced our understanding on the cellular interactions and molecular mechanisms directing successful axon regeneration in the spinal cord (Summarized in Table 1). Yet much remains to be understood. Conditional, cell type-specific manipulations are imperative to dissect the contribution of the complex neural and non-neural cell responses to the lesion environment and axon regeneration. The transgenesis amenability that zebrafish offer and the recent technological advances that allow for conditional gene inactivation [126], cell type-specific inducible gene expression [127,128,129], optogenetic manipulations [46,106], single cell RNA sequencing [130], large-scale genetic lineage tracing [131] and advanced optical techniques for in vivo-mapping of mechanical tissue properties [132] provide an optimal platform to characterize cellular relationships and contribution of different factors to spinal cord regeneration. Furthermore, the zebrafish SCI larval model is amenable to small molecule in vivo screens [133], which in combination with high-throughput, automated imaging and analysis platforms [134], can greatly accelerate the identification of potential targets to foster functional recovery after SCI in non-regenerating vertebrates. Finally, interspecies comparison on the multi-omics scale will be instrumental to map the differences that hamper regeneration in mammals yet facilitate functional recovery in zebrafish and other regenerating vertebrates [88,135]. We expect that future findings will provide exciting insight on what enables some vertebrates to successfully regenerate their CNS axons and recover locomotor function after injury.

## Figures and Tables

**Table 1:** Growth-promoting or limiting contributions of different cells types to axon regeneration in mammals and zebrafish. (X) indicates inhibitory and (✓) promoting or non-inhibitory effect of the indicated cell type on axon regeneration. (?) indicates that the mechanism of action remains to be shown. Abbreviations: ERG, ependymo-radial glia; OPC, oligodendrocyte progenitor cell; ECM, extracellular matrix.

		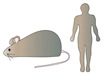		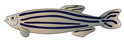		
Cell Type		Mechanisms of Growth-Modulation 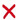 Inhibitory; 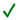 Promoting/Non-Inhibitory; 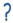 Unknown				Reference
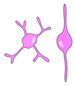	astrocyte glia/ ERG	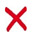	**glial scarring;** secretion of inhibitory ECM	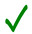	**glial bridging;** physical and trophic support (?)	[23,25,31,79,81,88]
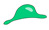	fibroblast	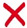	**fibrous scarring;** secretion of inhibitory ECM	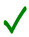	**no fibrous scarring;** secretion of growth- promoting ECM	[97,98,99,100,103,104,106]
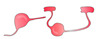	OPC/ oligodendrocyte	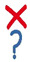	**growth cone entrapment;** low remyelination, secretion of inhibitory factors, formation of dystrophic endbulbs	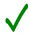	**no growth cone** **entrapment (?);** remyelination (?), secretion of growth- promoting factors (?)	[40,71,72,73,76]
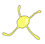	microglia	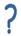	**prolonged inflammation;** secretion of pro-inflammatory cytokines	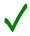	**limited inflammation;** phagocytosis, debris removal	[53,54,59]
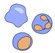	macrophage/ neutrophil/ T-cell	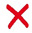	**prolonged inflammation;** secretion of pro-inflammatory cytokines	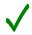	**limited inflammation;** phagocytosis, secretion of growth- promoting factors (?)	[24,55,56,57,60,61]
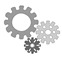	mechanical properties	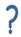	**mechanical barrier**	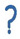	**growth-permissive** **mechanical tissue** **properties;** tissue stiffening	[112,114]
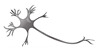	neuron-intrinsic factors	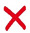	**limited growth capacity;** failure of growth cone formation and neurite extension	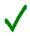	**elevated growth capacity (?);** upregulation of growth-promoting molecules	[22,28,34,36,115,116,117,118,119,120,121,122,123,124,125]

## Data Availability

Not applicable.
